# Mid- and Late-Life Chronic Kidney Disease Is Associated with Parkinson’s Disease, Not with an Increased Risk of Alzheimer’s Disease

**DOI:** 10.3390/jpm14060597

**Published:** 2024-06-03

**Authors:** Dong-Kyu Kim

**Affiliations:** 1 Institute of New Frontier Research, Division of Big Data and Artificial Intelligence, Hallym University College of Medicine, Chuncheon 24252, Republic of Korea; entkim@hallym.or.kr; Tel.: +82-33-240-5180; Fax: +82-33-241-2909; 2 Department of Otorhinolaryngology-Head and Neck Surgery, Chuncheon Sacred Heart Hospital, Hallym University College of Medicine, Chuncheon 24252, Republic of Korea

**Keywords:** kidney, dementia, Parkinson’s disease, Alzheimer’s disease, cohort studies

## Abstract

Chronic kidney disease (CKD) is strongly associated with dementia. However, its independent association with Alzheimer’s or Parkinson’s disease remains unclear. This study investigated the prospective association of patients with CKD aged ≥55 years with an increased risk of Alzheimer’s or Parkinson’s disease. We conducted a retrospective cohort analysis using a national cohort sample of approximately one million patients. Primary outcome indicators measured included incidence of all-cause dementia, Alzheimer’s disease, and Parkinson’s disease events using person-years at risk. The hazard ratio was adjusted using the Cox proportional hazards model. We included 952 patients without CKD and 476 with CKD over 55 years using propensity score matching. The CKD group exhibited higher incidences of all-cause dementia, Parkinson’s disease, and Alzheimer’s disease than the non-CKD group. Furthermore, the CKD group had an elevated risk of all-cause dementia and a significantly increased risk of Parkinson’s disease, especially among older women. Notably, the risk of Parkinson’s disease was higher within the first 3 years of CKD diagnosis. These findings emphasize the link between CKD in mid- and late-life individuals and a higher incidence of all-cause dementia and Parkinson’s disease rather than Alzheimer’s disease.

## 1. Introduction

The diagnostic criteria for chronic kidney disease (CKD) are kidney structural abnormalities or a glomerular filtration rate (GFR) < 60 mL/min/1.73 m^2^ that persists for more than 3 months. CKD also has a practical impact on health, and the older population has an increased CKD prevalence tendency [1]. GFR is an indicator of kidney filtration efficiency; thus, in patients with CKD, decreased GFR is associated with an increased risk of hospitalization, poor quality of life, and cognitive dysfunction [2,3,4]. Several studies have shown that CKD is a risk factor for diabetes, cardiovascular disease, and cognitive impairment [1,2,5]. Cognitive impairment is a common complication in patients with CKD [2]. Some studies have reported that cognitive function usually deteriorates in patients with CKD undergoing hemodialysis [6,7]. According to these studies, the prevalence of moderate-to-severe cognitive impairment in patients receiving hemodialysis ranges between 30% and 76%.

Dementia is a general term for a group of neurodegenerative diseases that affect thinking, memory, reasoning, personality, mood, and behavior and usually occur in the second half of life. In actual clinical practice, a significant number of patients with mild cognitive impairment progress to dementia, and according to research results, this progression rate is estimated at 1.9% per year [8,9]. A previous study reported that a decline in GFR was related to the subsequent development of all-cause dementia, specifically vascular dementia [10]. Furthermore, studies have reported no increased risk for Alzheimer’s-type dementia in patients with CKD [10,11,12]. However, a recent prospective cohort study identified an association between CKD and an increased risk of Alzheimer’s disease [13]. Moreover, several epidemiological studies have reported an increased risk of Parkinson’s disease in patients with CKD [14,15,16]. However, previous studies did not adjust independent variables that could affect the incidence or risk of neurodegenerative disease. Additionally, since neurodegenerative diseases mostly occur in elderly people, comparative research targeting more than middle-aged is needed rather than research targeting all age groups. Thus, our objective was to assess the future risk of these diseases in patients with CKD during mid- and late-life stages. Utilizing a nationwide cohort database in South Korea, we investigated the significant associations between CKD and all-cause dementia, Alzheimer’s disease, and Parkinson’s disease. To achieve this, we tracked the individual medical service history of more than 1 million Koreans. Additionally, we extracted a control group from the cohort dataset, ensuring sociodemographic matching, and compared the incidence and risk ratio of the primary end result between the target and control cohorts.

## 2. Materials and Methods

### 2.1. Ethical Approval and Data Availability

This study involved a nationwide population-based longitudinal analysis using a cohort dataset from the National Health Claims Database collected by the Korea National Health Insurance Service. The researchers received approval from the Ethics Committee of Hallym University Chuncheon Hospital (No. 2021-08-006). As the database provided non-identifiable secondary data during the review process, written consent was not required, and this requirement was waived. However, the detailed data profile of this study cannot be disclosed in accordance with the National Health Insurance Corporation’s privacy policy. Nevertheless, the authors have presented all of the data supporting the study results in the analysis section, and readers are informed that additional details can be provided upon request, subject to review.

### 2.2. Study Dataset, Design, and Participants

A cohort dataset consisting of 1,025,340 representative adults in Korea, equivalent to approximately 2.2% of the Korean population, was used in this study. Additionally, the cohort dataset has been shown to have “moderate to good” reliability in previous validation studies [17,18,19]. Among 1,025,340 patients, those with CDK were identified using diagnosis codes according to the International Classification of Diseases, 10th Revision (ICD-10). The index period for this study was set from 2003 to 2005. The CKD group included those who received two or more diagnoses of CKD (N18.1-5, N19) during hospitalization or outpatient treatment within this period. The inclusion criteria involved patients aged 55 years or older at the time of enrollment. The exclusion criteria for the CKD group included being diagnosed with dementia before being diagnosed with CKD during the index period. We also excluded cases diagnosed with CKD during the washout period. Propensity score-matched participants without a CKD diagnosis (2 participants without CKD per patient with CKD) were then randomly selected to form matched control participants (comparison group, non-CKD). These participants were matched with patients with CKD based on sociodemographic factors (age, sex, residential area, household income), comorbidities, and year of registration (year of CKD diagnosis). All cases of dementia-type diagnosis between the index period were excluded to increase the power of the comparison group. Through this process, 476 patients with CKD and 952 patients without CKD were finally selected for analysis in this study.

### 2.3. Primary Outcome

In this cohort observational study, the observation endpoint was defined as follows: death from all causes, diagnosis of dementia from all causes, Alzheimer’s disease (G30.0, G30.1, and G30.9), and Parkinson’s disease (G20) after the index period. Additionally, if a patient survived until the end of the cohort observation period (31 December 2013) without experiencing any events, the participant’s observation point ended after this time. Based on this observation period, the incidence rate and hazard ratio (HR) of the primary outcome in patients diagnosed with CKD and control participants were compared using the person-year risk method. Therefore, to apply the person-year risk method, we calculated the period between the date a patient was first enrolled and the end of observation for each patient individually.

### 2.4. Independent Variables

Table 1 summarizes the sociodemographic characteristics of the patients included in each group in this study, such as sex, age, place of residence, household income, and comorbidities. We matched variables by dividing them into two or three subgroups: male vs. female; age: 55–64, 65–74, and ≥75; income: bottom 30%, median 30.1–69.9%, and more than 70% of median; residential area: 1st-tier region (largest metropolitan area), 2nd-tier region (other metropolitan areas), and 3rd-tier region (small cities and rural areas); and comorbidities: Charlson Comorbidity Index (score: 0, 1, and 2).

### 2.5. Statistical Analyses

In this study, we divided the number of patients with all-cause dementia, Alzheimer’s disease, and Parkinson’s disease by the number of person-years at risk (per 1000 person-years) to calculate the incidence rate. Additionally, to assess whether CKD may increase the risk of specific diseases, HRs expressed as 95% confidence intervals (CIs) adjusted for other independent variables were calculated. In this case, we used Cox proportional hazards regression analysis. Finally, the specific disease-free survival rate of patients with CKD during the observation period was calculated using the Kaplan–Meier method. We used R version 4.0.0 software (R Foundation for Statistical Computing, Vienna, Austria) for all the statistical analyses in this study, and significance was set at a two-sided *p* value of 0.05.

## 3. Results

During the 10-year follow-up period, we evaluated the incidence of all-cause dementia, Alzheimer’s disease, and Parkinson’s disease between patients with CKD aged ≥55 years and the comparison (non-CKD) groups. Table 1 presents the characteristics of the selected cohort populations for the CKD and control groups. No significant differences were observed in each independent variable, including sex, age, residential area, household income, or comorbidities, between the groups. Additionally, to confirm the effectiveness of propensity scoring matching, we analyzed the balance plot of the five variables before and after propensity score matching. Similar distributions were observed in the balance plots of the two groups. Collectively, these findings imply that each independent variable was appropriately matched.

In patients with CKD aged over 55 years, we examined a total of 2778.2 person-years for all-cause dementia (8061.7 person-years in comparison), 2816.8 person-years for Alzheimer’s disease (8157.1 person-years in comparison), and 2780.8 person-years for Parkinson’s disease (8169.2 person-years in comparison). Additionally, we observed that the incidences of all-cause dementia, Alzheimer’s disease, and Parkinson’s disease per 1000 person-years were 9.36, 1.42, and 4.32, respectively, in the CKD group compared with 6.20, 1.23, and 1.10 in the control group (Table 2).

Furthermore, we analyzed the HR for incident all-cause dementia, Alzheimer’s disease, and Parkinson’s disease using univariate and multivariate Cox regression models (Table 2). After adjusting for all independent variables, we observed that patients with CKD aged ≥55 years showed a significantly increased risk ratio of the subsequent development of all-cause dementia and Parkinson’s disease events (adjusted HR = 2.01 [95% CI: 1.24–3.25] and adjusted HR = 3.98, [95% CI: 1.65–9.62]); however, no significant difference was observed in the risk ratio between CKD and Alzheimer’s disease.

We performed subgroup analyses to examine the association between patients with CKD aged ≥55 years and the development of Parkinson’s disease according to age and sex. After adjusting for other variables, the patients with CKD aged ≥55 years showed higher prospective development of Parkinson’s disease in women (adjusted HR = 4.33, 95% CI 1.02–18.32) than in men (adjusted HR = 3.94, 95% CI 1.28–12.09) (Table 3).

In addition, we observed that the adjusted HR for developing Parkinson’s disease in patients with CKD aged ≥55 years was higher with increasing age (adjusted HR = 3.91, 95% CI 1.15–13.27 in the 65–74 age category and adjusted HR = 18.05, 95% CI 1.17–278.62 in the ≥75 age category) (Table 4).

**Figure 1:**
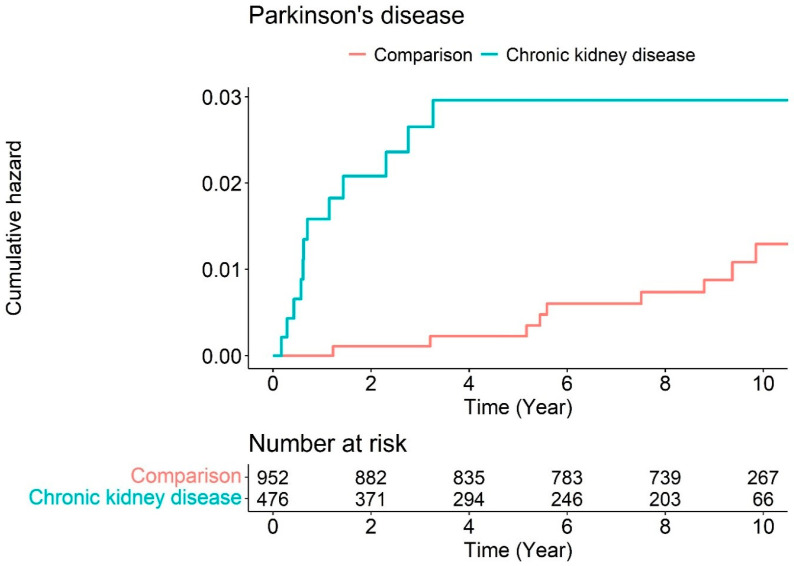
Cumulative hazard plot of Parkinson’s disease events between chronic kidney disease and comparison during the follow-up period.

Figure 1 illustrates the Kaplan–Meier survival curves with log-rank tests for the cumulative hazard plot of Parkinson’s disease events between the comparison and CKD groups. The results of the log-rank test indicated that patients diagnosed with CKD aged ≥55 years developed Parkinson’s disease more frequently than those not diagnosed with Parkinson’s during the 10-year follow-up period.

In the HR analysis over time, the risk of disease development in patients with CKD changed in a time-dependent manner. The risk for Parkinson’s disease after CKD development was relatively higher within the first 3 years, and the adjusted HR value gradually declined throughout the follow-up period (Table 5). This finding indicates that the association between CKD and Parkinson’s may not be temporal.

## 4. Discussion

CKD is an important risk factor for cognitive impairment owing to its vascular and metabolic factors. In this population-based longitudinal study, we examined the association between CKD and an increased risk of all-cause dementia, Alzheimer’s disease, and Parkinson’s disease using a cohort database of 1,025,340 South Korean patients. Our findings demonstrated that patients with CKD aged over 55 years experienced a significantly increased incidence and risk ratio of all-cause dementia and Parkinson’s disease; however, no relationship was detected between CKD and the incidence of Alzheimer’s disease. Notably, particularly in Parkinson’s disease, we observed that certain variables such as female sex and increasing age had a relatively higher risk ratio in patients with CKD aged over 55 years. Moreover, the risk of Parkinson’s disease in patients with CKD was relatively higher within the first 3 years after CKD diagnosis and gradually decreased over time but remained significant. This observation indicates that the association between CKD and Parkinson’s may not be temporal.

The etiology of CKD involves a complex interplay of various factors that contribute to the progressive loss of kidney function [20]. Primary causes include diabetes mellitus, where high blood sugar levels damage kidney blood vessels; hypertension, which impairs kidney function by damaging blood vessels; and glomerulonephritis, an inflammation of the glomeruli. Additionally, polycystic kidney disease, a genetic disorder characterized by kidney cysts, obstructive uropathy, blockages in the urinary tract, chronic infections, autoimmune diseases like lupus, nephrotoxic medications, and vascular diseases such as atherosclerosis all contribute to CKD. These factors, either alone or in combination, lead to the gradual decline in kidney function characteristic of CKD. Persistent kidney injury and diminished renal function also can negatively impact the function and structure of the kidneys, brain, gut, lungs, heart, and immune system [20,21,22]. Moreover, the cause of cognitive impairment in patients with CKD is multifactorial and includes impaired clearance of uremic metabolites, depression, sleep disturbance, and anemia [5]. However, the predominant pathology underlying CKD and cognitive impairment association is excessive vascular disease. Thus, since most patients with CKD are older adults, they may be considered at risk of developing Alzheimer’s disease, predominantly affecting memory in its early stages. Several previous studies have demonstrated that patients with CKD have a considerable risk of developing cognitive impairment compared to the general population, specifically with lower GFR or the presence of albuminuria [23,24,25]. Other studies have also suggested that lowering blood pressure and reducing albuminuria using angiotensin-converting enzyme inhibitors or angiotensin receptor blockers can modestly delay cognitive decline [26,27]. Therefore, numerous studies have assessed the relationship between CKD and an increased risk of dementia or Alzheimer’s disease, yielding conflicting results [10,11,12,13,28,29]. Specifically, we believe that these inconsistencies may arise from variations in the target population (sample size, matching control issues, and follow-up time). This present study used a representative nationwide cohort dataset with a relatively long follow-up period. Additionally, we matched the important variables between the cohort and comparative groups. Moreover, traditional cardiovascular disease risk factors were controlled between the two groups: older age, hypertension, dyslipidemia, and diabetes. Despite the extensive sample size and prolonged follow-up period, our findings did not indicate an elevated risk of Alzheimer’s disease associated with CKD. The incidence of Alzheimer’s disease in patients with CKD was comparable to that in patients without CKD, considering similar demographics and comorbidity burdens. This implies that Alzheimer’s disease is not the main or primary factor contributing to the increased risk. Similar to our findings, one recent study conducted on community-dwelling adults revealed that while reduced kidney function was linked to higher levels of blood biomarkers related to dementia, it did not correlate with an elevated risk of developing dementia [30]. Another study also described that there was no significant association between CKD and dementia [31]. However, our findings revealed that patients with CKD were associated with increased Parkinson’s disease events, suggesting an association between the two diseases.

Parkinson’s disease is the second most prevalent neurodegenerative disease affecting middle-aged and older adults. It manifests through cardinal clinical symptoms, including resting tremors, rigidity, bradykinesia, and impaired gait and posture. Several factors, such as diabetes and hypertension, may be associated with an increased risk of developing Parkinson’s; nevertheless, old age is a clear risk factor. The incidence of Parkinson’s disease increases with age, and the average age of onset is estimated to be 60 years; only approximately 4% of patients are diagnosed before the age of 50 years [32,33]. Currently, the prevalence of CKD has also increased to approximately 40% in individuals over 60 years of age [1]. In addition to old age, uremic toxins, vascular changes, and metabolic acidosis in CKD could also affect the basal ganglia, contributing to the development of parkinsonism [15,34,35]. Despite sharing common risk factors, previous studies present conflicting results. Several cohort studies have shown that patients with CKD have a higher risk of subsequently developing Parkinson’s disease than control groups [14,15,16]. Conversely, a recent study advocated that no significant correlation was observed between these two conditions [36]. However, this study also described an increased likelihood of developing Parkinson’s in patients with specific lifestyles and comorbidities, such as rural residents, normal weight, and hyperglycemia. Our comprehensive, nationwide, population-based study revealed that patients with CKD aged ≥55 years exhibited an elevated risk for the development of Parkinson’s compared to the non-CKD population; in particular, women and older individuals showed a higher risk. We also found a higher risk of Parkinson’s disease during the first 3 years after CKD diagnosis than during the subsequent period.

However, this study had some notable limitations. First, our cohort database did not contain details regarding CKD severity, such as GFR values. Based on the new guidelines, CKD is classified into five stages according to the GFR value [37]. Contrary to our findings, a previous cohort study from China showed that a lower baseline GFR was associated with a higher risk of all-cause incident dementia and Alzheimer’s disease. Thus, if CKD is defined using GFR values rather than ICD-10 diagnostic codes in the South Korean dataset, patients with CKD may be at an increased risk of developing Alzheimer’s disease. Second, the dialysis modality is a critical risk factor for the development of cognitive impairment in patients with CKD. Generally, peritoneal dialysis may involve much gentler hemodynamic shifts than hemodialysis. Thus, peritoneal dialysis theoretically causes reduced and less severe instances of brain injury in patients with CKD. For these reasons, dialysis type is an important covariant; however, our cohort database does not provide this information because of privacy concerns. Therefore, this bias could have distorted the true relationship between this study’s variable and its outcome, leading to inaccurate conclusions. Third, owing to our study’s observational and retrospective nature, we could not confirm whether our novel findings represent a causal link or a temporal incident event between the two diseases. Moreover, the mechanisms underlying the association between CKD and all-cause dementia, Parkinson’s disease, or Alzheimer’s disease remain unexplored. Finally, since family history, genetic predisposition, and radiographic findings based on magnetic resonance imaging often affect the potential development of Alzheimer’s disease, Parkinson’s disease, and vascular dementia, investigating the relationship between CKD and an increased risk of dementia is important. Thus, our findings have a risk of confounding bias. However, to overcome these limitations, we enrolled only patients aged 55 years or older and included a washout period of 1 year in this study. Moreover, we matched the CKD and comparative cohort groups using propensity scores based on several important independent covariates and had a relatively long follow-up period. Furthermore, to validate our findings, we analyzed the HRs over time, revealing that the significantly increased HRs of Parkinson’s disease events in mid- and late-life patients with CKD remained constant throughout the 11-year follow-up period. Therefore, future clinical and laboratory studies investigating a wider range of factors and diagnostic criteria are required to provide additional evidence for the association between CKD and dementia.

In conclusion, our study assessed whether CKD in mid- and late-life patients influences the subsequent development of all-cause dementia, Alzheimer’s disease, and Parkinson’s disease in mid- and late-life patients. We observed that CKD in mid- and late-life patients is associated with an increased risk of developing all-cause dementia and Parkinson’s disease, with the risk being greater in women and older patients. Additionally, CKD in mid- and late-life patients had no association with the risk of developing Alzheimer’s disease. Further studies are required to elucidate the underlying pathophysiological mechanisms. Therefore, clinicians should carefully monitor mid- and late-life patients with CKD and implement specific precautions to reduce the risk of developing dementia, particularly in those with Parkinson’s disease.

## Figures and Tables

**Table 1:** Characteristics of the study participants.

Variables	Comparison (*n* = 952)	CKD (*n* = 476)	*p* Value
**Sex**			1.000
Male	540 (56.7%)	270 (56.7%)	
Female	412 (43.3%)	206 (43.3%)	
**Ages (years)**			1.000
55–64	406 (42.6%)	203 (42.6%)	
65–74	350 (36.8%)	175 (36.8%)	
≥75	196 (20.6%)	98 (20.6%)	
**Residence**			1.000
Seoul	200 (21.0%)	100 (21.0%)	
Second area	236 (24.8%)	118 (24.8%)	
Third area	516 (54.2%)	258 (54.2%)	
**Household income**			1.000
Low (0–30%)	214 (22.5%)	107 (22.5%)	
Middle (30–70%)	286 (30.0%)	143 (30.0%)	
High (70–100%)	452 (47.5%)	226 (47.5%)	
**CCI**			1.000
0	302 (31.7%)	151 (31.7%)	
1	300 (31.5%)	150 (31.5%)	
≥2	350 (36.8%)	175 (36.8%)	

CKD: chronic kidney disease; comparison: participants without CKD; Seoul: largest metropolitan area; second area: other metropolitan cities; third area: other areas; CCI: Charlson Comorbidity Index.

**Table 2:** The incidence and the risk of all-cause dementia, Parkinson’s disease, and Alzheimer’s disease between CKD and non-CKD groups.

Variables	N	Case	Person-Year	Incidence	Unadjusted HR (95% CI)	Adjusted HR (95% CI)	*p* Value
All-cause dementia							
Comparison	952	50	8061.7	6.20	1.00 (ref)	1.00 (ref)	
CKD	476	26	2778.2	9.36	1.74 (1.08–2.81) *	2.01 (1.24–3.25) **	0.005
Parkinson’s disease							
Comparison	952	9	8157.1	1.10	1.00 (ref)	1.00 (ref)	
CKD	476	12	2780.8	4.32	3.63 (1.52–8.70) **	3.98 (1.65–9.62) **	0.002
Alzheimer’s disease							
Comparison	952	10	8160.2	1.23	1.00 (ref)	1.00 (ref)	
CKD	476	4	2816.8	1.42	1.22 (0.38–3.94)	1.31 (0.40–4.24)	0.656

CKD: chronic kidney disease; HR: hazard ratio; CI: confidence interval. * *p* < 0.05, ** *p* < 0.010.

**Table 3:** Hazard ratios of Parkinson’s disease by sex between CKD and non-CKD groups.

Sex	Male		Female	
	Comparison	CKD	Comparison	CKD
Parkinson’s disease				
Unadjusted HR (95% CI)	1.00 (ref)	3.30 (1.10–9.95) *	1.00 (ref)	4.00 (0.95–16.79)
Adjusted HR (95% CI)	1.00 (ref)	3.94 (1.28–12.09) *	1.00 (ref)	4.33 (1.02–18.32) *

CKD: chronic kidney disease; HR: hazard ratio; CI: confidence interval. * *p* < 0.05.

**Table 4:** Hazard ratios of specific diseases by age between non-CKD and CKD groups.

Ages	55–64		65–74		≥75	
	Comparison	CKD	Comparison	CKD	Comparison	CKD
Parkinson’s disease						
Unadjusted HR (95% CI)	1.00 (ref)	2.56 (0.51–12.94)	1.00 (ref)	3.54 (1.06–11.84) *	1.00 (ref)	8.42 (0.87–81.90)
Adjusted HR (95% CI)	1.00 (ref)	2.80 (0.54–14.45)	1.00 (ref)	3.91 (1.15–13.27) *	1.00 (ref)	18.05 (1.17–278.62) *

CKD: chronic kidney disease; HR: hazard ratio; CI: confidence interval. * *p* < 0.05.

**Table 5:** Hazard ratios for incident Parkinson’s disease event in patients with CKD by time since CKD diagnosis.

Time (Year)	Number of Parkinson’s Disease		Adjusted HR (95% CI)
	Comparison	CKD	
1	0	7	Not applicable
2	1	9	20.23 (2.56–159.87) **
3	1	11	25.14 (3.24–195.03) **
4	2	12	14.35 (3.20–64.32) ***
5	2	12	14.35 (3.20–64.32) ***
6	5	12	6.10 (2.14–17.40) ***
7	5	12	6.10 (2.14–17.40) ***
8	6	12	5.28 (1.97–14.17) ***
9	7	12	4.73 (1.84–12.14) **
10	9	12	3.98 (1.65–9.62) **
11	9	12	3.98 (1.65–9.62) **

CKD: chronic kidney disease; HR: hazard ratio; CI: confidence interval. ** *p* < 0.010, and *** *p* < 0.001.

## Data Availability

The datasets generated and/or analyzed in the current study are not publicly available owing to the policy of the Korea National Health Insurance Service, but are available from the corresponding author upon reasonable request.
